# What do we really know about age-related stereotypes and well-being of older adults? A commentary on the state of the art

**DOI:** 10.3389/fpsyg.2024.1358403

**Published:** 2024-05-13

**Authors:** Pauline Rasset, Jessica Mange, Maria Augustinova

**Affiliations:** ^1^Univ Rennes, Université Rennes 2, LP3C (Laboratoire de Psychologie: Cognition, Comportement, Communication)-UR1285, Rennes, France; ^2^Laboratoire de Psychologie de Caen Normandie (LPCN UR7452), Psychology Department, Université de Caen Normandie, Caen, France; ^3^Centre de Recherche sur les Fonctionnements et Dysfonctionnements Psychologiques (CRFDP UR7475), Université de Rouen Normandie, Rouen, France

**Keywords:** age-related stereotypes, self-directed stereotypes, stereotype threat, internalization of stereotypes, hedonic well-being, eudaimonic well-being

## Abstract

There is a considerable body of literature on harmful consequences of age-related stereotypes—including consequences on physical and mental health. However, this commentary critically argues that the current state of the art disregards consequences of these stereotypes specifically for the well-being of older adults (i.e., outcome that is not to be confounded with mental health). To this end, the content of age-related stereotypes and the mechanisms through which they operate on physical and mental health are first outlined. The commentary then focuses on the very scarce evidence documenting how and when the well-being of older adults (as assessed directly and not as inferred from other indicators) is influenced by self-directed stereotypes. After setting out possible ways well-being may be involved in the relationship between self-directed stereotypes and physical and mental health of older adults, the present commentary argues that a better understanding of well-being would benefit strategies targeting the reduction of age-related stereotypes. Overall, this commentary on the state of the art highlights that future research is still needed to better understand both the direct and indirect relationships between age-related stereotypes and well-being that is not reducible to positive experiences of life (or hedonic well-being) but also comprises an eudaimonic component.

## 1 Introduction

Currently, an average individual lives between 4 and 6 years longer than would have been expected 20 years ago (see life expectancy at birth; Institut National de la Statistique et des Etudes Economiques (INSEE,2020); World Health Organization (WHO, 2020a). Additionally, individuals over 65 make up 9% of the global and 19% of the European population today, and these percentages are expected to increase considerably between now and 2050 (INSEE, 2020). Since we undeniably live in an aging society, the current prevalence of *ageism*—initially defined as prejudice and discrimination against older individuals (Butler, 1969)—might come as a surprise. But while at the age of 65, individuals are expected to live for approximately 19.2 more years (see life expectancy at 65 years in 2021; Eurostat, 2023b), their *healthy* life expectancy is only half of that (see healthy life expectancy at 65 years in 2021; Eurostat, 2023a). As a result, older people are increasingly being described not only as vulnerable (i.e., benevolent ageism), but also as a burden for society (i.e., hostile ageism; Gans et al., 2023). Needless to say, both of these narratives—particularly salient during the COVID-19 pandemic (e.g., Cohn-Schwartz and Ayalon, 2021)—have devastating consequences for both the physical and mental health of older adults. Although authors have been pointing to these consequences for several decades (e.g., Levy et al., 2002), it is only recently that the United Nations (UN) has called for progress in addressing ageism and, more generally, factors that predict the health and well-being of older adults (WHO, 2020b). While these initiatives are supported by the existing literature tackling the influence of age-related stereotypes on physical and mental health, this literature largely disregards a somewhat obvious relationship between age-related stereotypes and the well-being of older adults, meaning that it is therefore largely unknown.

**Figure 1:**
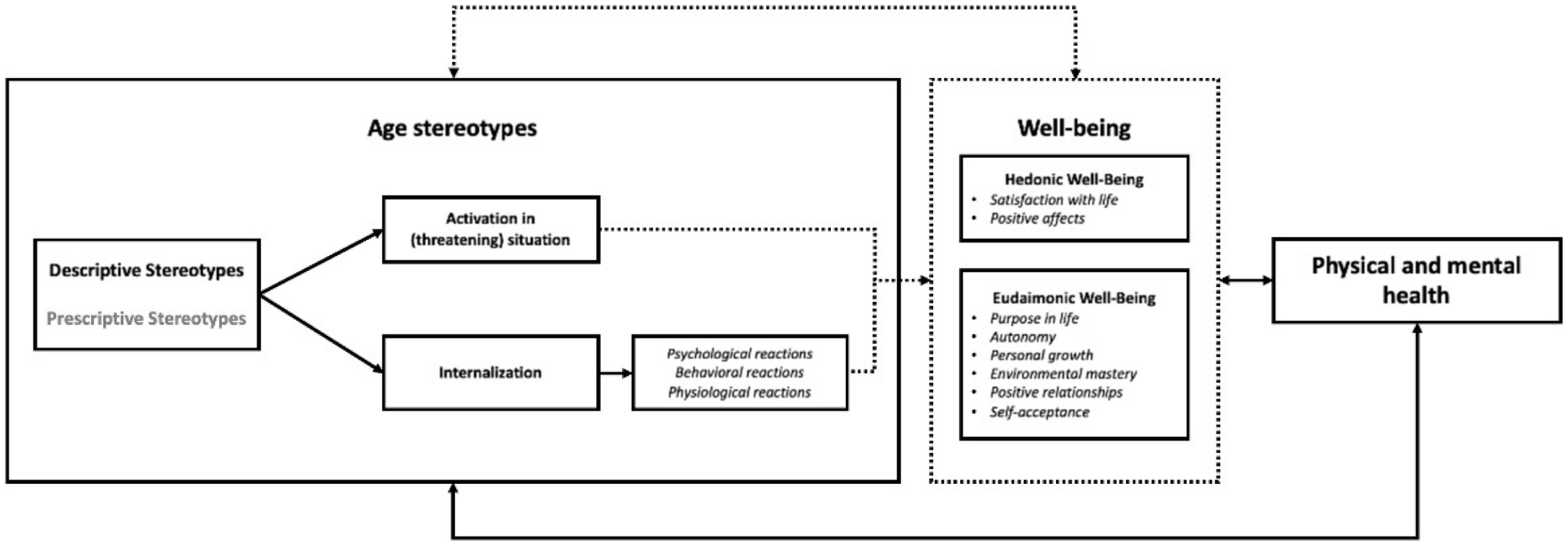
Schematic representations of current knowledge on (possible) relationships between age stereotypes and well-being. Dotted lines (i.e., relationships between age stereotypes and well-being, and the mediating role of well-being) and grey police represent areas in which further research and a more accurate understanding of those relationships are needed.

Age-related stereotypes constitute a core cognitive component of ageism. As such, they predict prejudice and discrimination against older individuals by younger adults (i.e., as an outgroup). However, it is important to underline that this latter outgroup also constitutes a prospective ingroup, meaning that those who held negative (other-directed) age-stereotypes (and potentially engage in prejudice and discrimination against older adults) later become a *target* of these very same stereotypes. This additionally implies that older adults who hold negative stereotypes of older age are both the *source* and *target* of these stereotypes, thus leading to quite particular but also potent mechanisms through which self-directed age-related stereotypes operate and influence not only physical and mental health of older adults but also their well-being (see Figure 1). Indeed, although health and well-being are both considered as pillars of healthy aging (WHO, 2020b), the present commentary on the current state of the art argues that the negative influence of self-directed age stereotypes on well-being specifically (as opposed to on the physical and mental health of older adults; e.g., Chang et al., 2020) has as yet received far too little attention.

Indeed, well-being has traditionally been conceptualized as reflecting the absence of mental health difficulties (i.e., namely the absence of depression, anxiety and stress). In contrast to this latter view, which actually disregards the “well” component of well-being, it is now instead considered to reflect a person’s self-reported happiness and satisfaction with his/her quality of life (e.g., Green et al., 2021). It is, however, important to understand that this—perhaps qualitatively different—view of well-being is still somewhat restrictive in the light of recent research arguing an additional and equally important component of well-being exists (Butler and Kern, 2016; Ryff et al., 2021a). A further aim of the present commentary on the current state of the art is therefore to reaffirm the need to consider well-being as a multifaceted and complex phenomenon that goes beyond life-satisfaction (or the hedonic conception of well-being; Pancheva et al., 2021). To this end, the content of age-related stereotypes and the general mechanisms driving their influence on physical and mental health are first outlined. The commentary then focuses on the literature dealing specifically with the influence of age-related stereotypes on well-being and its components and with processes underlying this influence. Following this literature review, this commentary then discusses the challenges to achieving a better understanding of the relationship between age-related stereotypes and well-being in older-adults, and the way this latter relationship is linked to both physical and mental health.

In order to indicate more informed directions for future studies and applied interventions, the aforementioned review of the literature on the influence of age-related stereotypes on well-being (see sections 3 “Age-related stereotypes and their influence on older adults” and 4 “Unresolved issues surrounding the role of older adults’ well-being”) is a narrative review (see Baumeister and Leary, 1997; see also Ioannidis, 2016; Greenhalgh et al., 2018 for arguments in favor of this type of review). Indeed, in line with the idea that narrative reviews specifically aim to provide interpretations and critiques (Greenhalgh et al., 2018), the primary goal of this critical review of the current state of the art was to look in much greater detail at the overlooked theoretical and methodological issues regarding the relationship between age-related stereotypes and the well-being of older adults, instead of comprehensively summarizing the data documenting this relationship (as conventional systematic reviews would do) or assessing its strength (as a meta-analytic form of literature review would do).^1^

To perform this narrative review, a literature search was conducted in the CAIRN, PsycArticles, Sage Journals, Science direct, and Web of Science databases. The search query included synonyms of: age-related stereotypes (e.g., stereotypes on old age), self-stereotypes and related concepts (e.g., self-perceptions of aging, views on aging), self-oriented impact of stereotypes (e.g., internalization of stereotypes, stereotype threat), health, hedonic well-being (e.g., satisfaction with life), eudaimonic well-being (e.g., purpose in life). To be considered, studies had to be published in English or French and report descriptions of the impact of self-oriented stereotypes on health and/or on well-being. Additional research was extracted from a search of the references in the identified articles. Since our aim was not to conduct a systematic but a critical literature review, we iteratively updated our literature search to ensure we selected evidence judiciously and in a way that accurately addressed the issues relating to how self-oriented stereotypes impact the well-being of older adults.

## 2 Age-related stereotypes and their influence on older adults’ physical and mental health

### 2.1 The content of the stereotypes of old age

#### 2.1.1 Descriptive old age stereotypes

Age-related stereotypes are defined as cognitive structures embedding beliefs and expectations that people hold about different age stages (Cuddy and Fiske, 2005). The content of old-age stereotypes includes physical characteristics (e.g., unattractiveness), social status (e.g., retirement), behavioral tendencies (e.g., slowness; for a review, see Hummert, 2011). Overall, therefore, stereotypes of older age are not entirely negative (Hummert, 2011; Ng and Indran, 2022). The content of stereotypes varies as a function of several factors, including context (Casper et al., 2011), subtypes of “old age” (e.g., “grandmother” vs. “elder statesman”; see Cuddy and Fiske, 2005; Chasteen et al., 2022), life domains (e.g., family vs. health; Kornadt and Rothermund, 2011), characteristics of the targeted older person [e.g., gender, chronological age; (Kornadt et al., 2013; Kydd et al., 2018)], or of his/her perceiver [e.g., culture, age; (Löckenhoff et al., 2009; Kornadt et al., 2016; Ackerman and Chopik, 2021)]. To sum up, older adults are generally viewed as having more warmth-related (i.e., characteristics related to the intents of older adults) than competence-related characteristics (i.e., characteristics related to the ability to enact those intents; Fiske, 2018) (see the stereotype content model; Fiske et al., 2002; Cuddy et al., 2005; Fiske, 2017; Kuljian and Hohman, 2023).

Nevertheless, over the past two centuries, the old-age stereotypes expressed in various American media have become more negative (Ng et al., 2015) and negative stereotypes are likely to prevail (Kite et al., 2005), thereby potentially influencing age-related prescriptive stereotypes (Cohn-Schwartz and Ayalon, 2021).

#### 2.1.2 Prescriptive old-age stereotypes

Descriptive and prescriptive stereotypes are closely related. Prescriptive stereotypes refer to beliefs about how older adults should be and behave (De Paula Couto and Rothermund, 2022; De Paula Couto et al., 2022a,b). They serve a social function by enjoining older adults not to become a burden for society (De Paula Couto et al., 2022a; but see Kitayama et al., 2020 for cultural variations). As such, older adults are expected to disengage in three specific domains: succession (i.e., give up important positions for younger people), consumption (i.e., save resources), and identity (i.e., behave as is appropriate for their own age; North and Fiske, 2013a,b). They are also expected to remain active (e.g., to continue working after retirement or to get involved in associations). Finally, older adults are expected to be wise (e.g., be able to transcend their individual concerns by focusing on the common good) and behave in a dignified way (De Paula Couto and Rothermund, 2022). Older adults are rewarded when they comply with these prescriptions but severely punished when they do not (North and Fiske, 2013b). Nevertheless, we do not possess clear evidence for mechanisms underlying the effects of prescriptive stereotypes—especially those of self-directed prescriptive stereotypes—in the same way as we do for descriptive stereotypes, which are outlined below (see also Figure 1).

### 2.2 General mechanisms through which descriptive old-age stereotypes burden the physical and mental health of older adults

The ample social psychological literature agrees that the effects of descriptive stereotypes on the physical and mental health of older adults are underpinned by two distinct general mechanisms: the activation of stereotypes by the situation, also called stereotype threat, and the internalization of stereotypes (see, e.g., Chasteen et al., 2022).

#### 2.2.1 Stereotype threat

This line of research shows that negative age-related stereotypes can become self-fulfilling prophecies hampering the performance of older adults.^2^ For instance, during clinical evaluations older adults are likely to be concerned that their behavior (e.g., performance on memory tests) could confirm the negative stereotype (e.g., aging involves dementia) that they hold about themselves (Barber, 2017); (e.g., Fresson et al., 2017). Accordingly, Mazerolle et al. (2016) observed that participants who felt highly threatened performed worse than low-threat participants on standardized memory tests (i.e., MOCA and MMSE). This impaired performance was such that 40% of the former participants met the clinical criteria for mild cognitive impairment (MCI) on these tests (as compared to 10% of the latter; for research outside of laboratory settings, see also Ben-David et al., 2018; Phibbs and Hooker, 2018; Gauthier et al., 2020). One major implication is that, instead of reflecting the older adults’ actual memory capacities, the MCI diagnosis instead reflects (at least partly) old-age stereotype threat and the behavioral confirmation to which it gives rise (Régner et al., 2016; for a meta-analysis, see Armstrong et al., 2017).

Because negative stereotypes concerning the incompetence of older adults cut across a wide range of domains (see the different life domains of age stereotypes; Kornadt and Rothermund, 2011), situations other than cognitive/memory assessment can also be threatening (Barber, 2020; Lamont et al., 2021). Several other age-based stereotype threat situations have been identified, including gait (Barber, 2020), driving (Chapman et al., 2016), or computer use (Mariano et al., 2020).

#### 2.2.2 Internalization of stereotypes

Self-directed age stereotypes are part of a broader construct, namely the self-perceptions of aging (i.e., the meaning of being old oneself; Kornadt et al., 2022).^3^ According to stereotype embodiment theory (Levy, 2009), people internalize the age-related stereotypes they encounter in their cultural environment from an early age. When these stereotypes become self-relevant (i.e., when people enter old age), these stereotypes turn into self-definitions (i.e., self-perceptions of aging) and exert an influence on their physical and mental health (see also Kornadt and Rothermund, 2012). Various meta-analyses have consistently reported that negative views of aging predict poorer health outcomes as measured by longevity, quality of life, social relationship, healthy behavior, mental illness, cognitive impairment, and physical illness (Westerhof et al., 2014, 2023; Chang et al., 2020; Hu et al., 2021; see also Tully-Wilson et al., 2021).

The influence of stereotypes on health operates along three pathways (Levy, 2009). First of all, there is the physiological pathway via which stereotypes engender a psychological stress which is reflected in a physiological stress response. For instance, C-reactive protein (i.e., a marker of cumulative stress-related inflammation) has been found to be a mediator of the influence of self-perceptions of aging on survival (Levy and Bavishi, 2018). Second, there is the behavioral pathway via which negative stereotypes suppress preventive and health-promoting behaviors. For instance, the influence of self-perceptions of aging on survival is mediated by a healthy lifestyle (Zhang et al., 2020) and more negative self-perceptions of aging predict unhealthy eating (Klusmann et al., 2019). Accordingly, older people with less positive self-perceptions of aging have been found to be more likely to develop obesity (Levy and Slade, 2019). Third, there is the psychological pathway via which negative stereotypes undermine psychological resources. For instance, people with more negative self-perceptions of aging have been found to be more likely to develop depression and anxiety (Freeman et al., 2016), and a decreased “will to live,” which directly reduces their probability of survival (Levy et al., 2002; Kotter-Grühn et al., 2009).

To sum up, this section has provided a brief summary of the impact of self-directed age-related stereotypes on the physical and mental health of older adults (for a complete recent review, see Rothermund and De Paula Couto, 2024), with the result that these latter outcomes can be more clearly distinguished from the central focus of the present literature review, namely older adults’ well-being.

## 3 Age-related stereotypes and their influence on older adults’ well-being

### 3.1 The multiple facets of well-being

There are conceptual and methodological challenges surrounding well-being and the way it is measured (see Ryff et al., 2021a). Indeed, well-being is a complex and multifaceted phenomenon (Butler and Kern, 2016; Ryff et al., 2021a). Well-being relates both for to the realization of personal potential (i.e., the eudaimonic conception) and positive life experiences (i.e., the hedonic conception; Pancheva et al., 2021). While research on well-being has long focused on the hedonic aspect (i.e., by focusing attention on happiness, satisfaction with life, positive emotions, and the absence of negative emotions), more recent research has stressed the need to consider its eudaimonic side (i.e., with attention being given to autonomy, personal growth, self-acceptance, purpose in life, environmental mastery, or positive relationships; Ryff and Keyes, 1995; Disabato et al., 2016; Ryff et al., 2021a).

Due to the high degree of inter-individual variability, the results of research on the impact of older age on well-being remain inconsistent (see Ryff et al., 2021a,b for an ample discussion). The impact of age on well-being results from various factors and not just their chronological age (Steverink, 2019). These include psychosocial factors, such as age-related stereotypes. Despite this, there are various reasons accounting for the small volume of research on the impact of stereotypes on well-being. First, the concept of well-being itself has rarely been measured directly. Instead, it is often inferred from other indicators, such as the absence of mental disorder (e.g., depression; Lu et al., 2010). For instance, a recent systematic review evaluated ten age stereotype-based interventions (Knight et al., 2022). It appears that three of them measured “psychological well-being” as an outcome measure, but two of them used depression scales. Two studies measured “generativity,” i.e., psychosocial features of people who consider it important to provide for the next generation (McAdams et al., 1993), or “self-esteem,” both of which are correlates, but not direct measures, of hedonic and eudaimonic well-being. Second, when well-being is measured directly, it almost always relates to the hedonic component of well-being (e.g., satisfaction with life and positive affects; Steverink et al., 2001; Zhang et al., 2018). Nevertheless, some studies do show an impact of stereotypes on well-being and these are reviewed below.

### 3.2 The impact of stereotypes on hedonic well-being

Research exists showing that ages stereotypes impact hedonic well-being. For instance, holding positive age-related stereotypes predicts better life satisfaction and more positive affects (Kornadt and Rothermund, 2011; Söllner et al., 2022). However, due to the measure employed, it is difficult to pinpoint the distinct influence of self-stereotyping independently of that of other closely-related processes—such as the stereotyping of other adults or age-independent self-perceptions—is challenging (see text footnote 3).

Nevertheless, research into stereotype threat provides some answers. Broadly speaking, stereotype threats impairs well-being (Lewis and Sekaquaptewa, 2016). In the workplace, age-based stereotype threat has negative implications for (hedonic) workplace well-being (von Hippel et al., 2019). Although this effect is particularly great for older workers, the entire sample of this study was under the age of 66, thus making it difficult to interpret the results in terms of a general impact of age-based stereotype threat on the well-being of older adults.

There are variations according to the type of prescriptive stereotype. It appears that prescriptive stereotypes of active aging are positively related to hedonic well-being (i.e., life satisfaction), with the reverse being observed for prescriptive stereotypes of altruistic disengagement (De Paula Couto et al., 2022a). In sum, this research suggests that self-directed age-related stereotypes do not always impair hedonic well-being, depending on the nature of the stereotypes (i.e., whether they value or disparage older adults).

### 3.3 The impact of stereotypes on eudaimonic well-being

While these positive experiences of life have made it possible to draw at least some inferences about older adults’ hedonic well-being, the extent to which the realization of older adults’ personal potential (or eudaimonic well-being) is directly influenced by positive vs. negative stereotypes is still largely unknown. There are nevertheless some studies showing effects similar to those on hedonic well-being. Thus, holding positive age-related stereotypes also predicts having a meaning in life (Söllner et al., 2022). In the workplace, age-based stereotype threat also has negative implications for eudaimonic workplace well-being, impairing functioning and future time perspective in relation to work (Manzi et al., 2019). Again, this study concerned older workers who were not necessarily elderly adults.

One study suggests that self-directed (rather than other-directed) age stereotypes play an important role in eudaimonic well-being. Kim et al. (2019) investigated the impact of perceived ageism on the sense of purpose in life. They revealed that this effect was fully mediated by self-perceptions of aging. All these results are promising but limited because they are few in number and do not concern all the sub-dimensions of well-being.

It appears crucial to make at least some distinction between the two types of well-being. For instance, when discussing prescriptive stereotypes, we referred to research showing a beneficial impact of prescriptions of activity on hedonic well-being (De Paula Couto et al., 2022a). Other research suggests that such stereotypes may have a harmful effect on eudaimonic well-being (Kitayama et al., 2020). More precisely, it appears that the prescription of activity varies according to the culture (i.e., true for Western cultures but not Eastern ones). Thus, failure to meet these cultural expectations concerning activity—as well as those relating to independence and positivity—could explain why western older adults (but not eastern older adults) experience a decline in eudaimonic well-being during the later stage of their lives (Kitayama et al., 2020).

## 4 Unresolved issues surrounding the role of older adults’ well-being

The purpose of this commentary on the current state of the art is to argue that well-being in older adults is not simply another outcome of self-directed age-related stereotypes, alongside other variables such as those related to mental health. Instead, one of the primary objectives of this commentary is to emphasize that older adults’ well-being is probably fundamental to the way in which these stereotypes influence both their physical and mental health (and therefore *healthy* life expectancy). In this section, we will discuss research that supports this idea that well-being is bound up with other outcomes of self-directed age-related stereotypes. First, we will explore the potential implications of well-being for the known moderators of these stereotypes. Second, we will investigate how well-being may potentially mediate the way these stereotypes influence the health of older adults. Finally, we will address some limitations regarding these various processes.

### 4.1 Moderators of age-related stereotypes: how do they impact well-being?

Not all individuals experience the harmful effects of stereotyping depicted above (see sections 2.2.1 and 2.2.2.). Indeed, some older adults are more successful than others in developing strategies to mitigate or even counteract the negative impact of old-age stereotypes, as the following examples show.^4^ First, some individuals seek to dissociate themselves from their age group by emphasizing a younger self-identity (Weiss and Lang, 2012; Weiss et al., 2013; Wurm et al., 2017; Weiss and Kornadt, 2018). Even without having to dissociate themselves from their group, older adults can protect their self-esteem by engaging in downward social comparison (i.e., comparison with worse-off others) resulting in better mental health (Stewart et al., 2013). Second, just as the endorsement of negative stereotypes is associated with negative outcomes, the endorsement of positive stereotypes is likely to be associated with positive outcomes (Wurm and Schäfer, 2022). Accordingly, research has shown that positive self-perceptions of aging positively influence preventive health behaviors (Levy and Myers, 2004) and their consequences, including a reduction in the risk of developing later obesity (Levy and Slade, 2019). Third, research suggests that older adults who adopt a non-essentialist view experience better memory performance and less stress in stereotype threat situations than older adults who adopt an essentialist view of aging (Weiss, 2018; for a review, see Weiss, 2022). These findings suggest that instilling a growth mindset (i.e., a lay theory about the malleability of individual characteristics; for a review, see Chasteen et al., 2022) could help counter negative old-age stereotypes. Finally, older adults also cope with age-related stereotypes by countering them directly. For instance, the existing evidence suggests that the prevalence of various psychiatric disorders is lower among military veterans who resist negative age stereotypes (Levy et al., 2014b). Resistance to stereotypes is rooted in the ability to associate old age with positive concepts and to overcome negative associations (Gonsalkorale et al., 2014). Engagement in active coping (i.e., taking action to eliminate or reduce the stressor) reduces the impact of negative stereotypes of old-age on psychiatric conditions (Levy et al., 2019).

Although this list of factors and/or strategies that mitigate the negative impact of age-related stereotypes on physical and mental health is far from being complete, it nevertheless shows that these strategies have considerable potential for protecting and even improving older adults’ well-being (see text footnote 4). There is, however, little evidence illustrating this idea directly. For instance, perceiving the aging experience as continuous growth predicts life satisfaction and positive affect. Moreover, the more positive age-related changes that are perceived, the fewer the perceived negative age-related changes and the more expansive the view of the future and the better psychological well-being are likely to be (Brothers et al., 2016). By contrast, older subjective age is associated with poorer life satisfaction and higher negative affect (Westerhof and Barrett, 2005), with positive attitudes towards aging moderating this negative impact (Mock and Eibach, 2011). In the same way, perceiving the aging experience as physical decline predicts less positive and more negative affect (Steverink et al., 2001). In sum, any given factor that influences the impact of age-related stereotypes should, in principle, influence not only the hedonic aspect (i.e., with attention focused on happiness, satisfaction with life, positive emotions, and the absence of negative emotions) of well-being, but also its eudaimonic component. However, future research needs to tackle this unresolved issue directly.

It is also noteworthy that the aforementioned self-defense strategies have been incorporated into interventions and programs designed to lower the burden of age-related stereotypes outside of the lab. For instance, intervention exist that try to change descriptive stereotypes. Typically, participants exposed to implicit positive age stereotypes are found to be more likely to display positive age stereotypes, positive self-perceptions of aging, and improved physical function than participants exposed to explicit positive age stereotypes (Levy et al., 2014a). There are also interventions targeting the pathways of internalized stereotypes (for a full review, see Steward, 2022) or age-related stereotype threat (for a meta-analysis, see Liu et al., 2021). However, as far as we are aware, there is still some uncertainty regarding the specific benefits of these interventions for enhancing the well-being of older adults.

### 4.2 Evidence for (and against) the mediating role of well-being

The above-mentioned research suggests that well-being—not just physical and mental health—may be impeded by age-related stereotypes. Since well-being predicts a range of health variables (for a review, see Ryff, 2018) that are also impacted by age-stereotypes, well-being may play a role in mediating the influence of these stereotypes on health. For instance, internalized age stereotypes influence longevity (Wurm and Schäfer, 2022), as does well-being (Boyle et al., 2009). Internalized age stereotypes influence physiological stress response (Levy and Bavishi, 2018) in the same way as well-being (Friedman and Ryff, 2012). Internalized age stereotypes predict biomarkers of Alzheimer’s disease (Levy et al., 2016), just as well-being does (Boyle et al., 2010). Again, these research examples make it highly plausible that well-being helps mediate the influence of the stereotypes on both mental and physical health. However, the kind of well-being involved here (hedonic vs. eudaimonic) is as yet unknown—opening avenues for future research.

Moreover, both internalized age stereotypes and well-being influence preventive health behaviors—albeit different ones (Levy and Myers, 2004; Kim et al., 2014). Thus, well-being may mediate the effects of internalized age stereotypes on health, at least via some of the pathways. However, the reverse may also be true, with well-being turning age stereotypes into something positive. Previous research has already documented how health and well-being can improve views of aging (for a review, see Kornadt et al., 2020). For instance, it is now acknowledged that life experiences shape what it means to age. As such, it is possible that the greater the well-being, the more positive the age stereotypes are. In line with this assumption, one study found that older adults who were enthusiastic volunteers (i.e., an activity known to give a greater sense of purpose to life and that is a dimension of eudaimonic well-being) had a better self-perception of aging (Huo et al., 2021). It is thus possible that fostering the well-being of older adults (and perhaps specifically eudaimonic well-being) might contribute to a virtuous circle by overcoming age stereotypes and their detrimental impacts. Again, more empirical evidence is needed to reliably answer these as yet unresolved issues.

### 4.3 Some (other) limitations and further complexities

One of the goals of the present commentary is to emphasize the fact that despite the wealth of literature on the consequences of age-related stereotypes (e.g., Rothermund and De Paula Couto, 2024 for recent reviews), and also on the consequences of age-related stereotypes of mental and physical health (e.g., Chang et al., 2020), little research, whether into stereotype threat or internalization, has as yet studied the direct impact of negative stereotypes of aging on the well-being of older adults. Far more research of this type is therefore needed and should make use of direct measures of well-being, conceptualized as a complex, multifaceted phenomenon encompassing both hedonic and eudaimonic components (Butler and Kern, 2016; Ryff et al., 2021a). This is especially true for research concerned with the implications of well-being for the impact of self-directed age stereotypes on health.

However, we believe that measuring self-stereotypes in such research also raises a number of problems. Indeed, it is difficult to separate the impact of self-stereotyping from other closely related processes, such as the stereotyping of other older adults or age-independent self-perceptions (see above and text footnote 3). Even so, there is experimental evidence of these self-stereotypes. Based on the assumption that age stereotypes are internalized across the life span and that they can operate even unconsciously, Levy and Leifheit-Limson (2009) demonstrated in an ingeniously designed study that older people primed with negative stereotypes underperformed on a stereotype-congruent task (e.g., Levy and Leifheit-Limson, 2009). More generally, one meta-analysis found that age priming had an impact on behavior, with negative having a greater effect than positive priming (Meisner, 2012). Nevertheless, to provide better evidence of the effect of self-stereotypes on well-being (and its dimensions), future research should address the issue of how to measure self-directed stereotyping.

This is all the more true with regard to the distinction between descriptive and prescriptive stereotypes. For the latter, the problem is rendered even more complex because there is less empirical evidence of the effects of prescriptive stereotypes than there is for those of descriptive stereotypes. Future research on the link between age stereotypes and the well-being of older adults therefore needs to consider the distinction between descriptive and prescriptive stereotypes. It also needs to look closely (and directly) at the influence of each of their sub-categories, as they might have contrasting impacts on hedonic and eudaimonic well-being (and therefore potentially explain the currently mixed results on the well-being of older adults).

We have primarily focused on the positive effects of well-being, reflecting the considerable body of evidence demonstrating its beneficial impacts—whether hedonic or eudaimonic—on various health outcomes (see Hernandez et al., 2018). Nonetheless, it seems reasonable to acknowledge that well-being may, to some extent also have adverse effects on health. For instance, research has demonstrated that positive emotions such as joy may encourage risk-taking behavior by fostering overconfidence (Koellinger and Treffers, 2015). Consequently, it is possible that hedonic well-being could lead to carelessness (e.g., the persistent feeling of being fine might discourage people from going for regular health checkups), thus potentially impairing the health of older adults. This aspect merits further attention.

## 5 Conclusion

A considerable body of literature has documented how stereotypes of old age constitute a societal, social, and individual burden. Some of this literature also specifically addresses the impact of these stereotypes on older adults’ physical and mental health. Despite this, the impact of old-age stereotypes on the well-being (i.e., *not* defined as the absence of mental health issues) of older adults and the more complex dynamics that this multifaceted outcome entails are still largely unknown. Consequently, future basic and applied research should study the direct impact of self-directed age stereotypes on the hedonic and eudaimonic well-being of older adults.

Indeed, we live in an aging society where, for example, the average age of the workforce is expected to increase still further. Therefore, the numbers of interventions and programs targeting age stereotypes and well-being is increasing. However, such studies are often conducted in isolation. As Steverink (2019) puts in: “Well-being is not only a desirable outcome, it also has been found to be an important predictor of all kinds of positive outcomes.” Despite this, well-being is rarely considered as an indicator of the effectiveness of stereotype-based interventions. Thus, future research should determine the extent to which well-being (and its subcomponents) can be predicted by age stereotypes (and their reduction) when both of these are measured directly. Moreover, the research discussed in this paper also clearly suggests that the well-being of older adults and initiatives undertaken to improve it would benefit greatly from a reduction in age-related stereotypes, an aim that could perhaps be achieved through future interventions and field programs.

## Author contributions

PR: Conceptualization, Writing – original draft, Writing - review & editing. JM: Funding acquisition, Supervision, Writing – original draft. MA: Funding acquisition, Supervision, Writing – original draft, Writing - review & editing.

## Footnotes

^1^It should be noted that the present paper does not argue against these two other types of reviews. Simply, as we argue bellow, more ample and more direct evidence is needed of the relationship between age-related stereotypes and well-being of older adults before the field can truly benefit from a systematic review or a metanalysis (Ioannidis, 2016; Greenhalgh et al., 2018).

^2^Although some controversy surrounds the robustness of the stereotype threat phenomenon in general (see, e.g., Zigerell, 2017), this does not apply to age-based stereotype threat.

^3^Recently, much effort has focused on delimiting related concepts such as views of aging (i.e., conceptions about older people), awareness of age-related changes (i.e., experiences that make people aware of having grown older), self-perceptions of aging or self-stereotypes (Wurm et al., 2017; Shrira et al., 2022). Although there are distinctions to be made between these psychological constructs, it is difficult to isolate their specific influence or infer their isolated causality because of their close relationships and the nature of the experimental designs used in research. Therefore, the term self-perception is used to cover all these concepts here.

^4^Several moderators that drive the internalization or dissociation of age-related stereotypes—consequently impacting health outcomes—have been identified (e.g., alternative age identities such as generation instead of chronological age). We have chosen to mention here only those that may have an impact on well-being (but for a comprehensive review, see Weiss and Kornadt, 2018).
